# Supplementary material for: Critical roles of MCM8 in meiotic recombination during mouse spermatogenesis
Source: bioRxiv. 2026 Mar 30:2026.03.28.714908. Preprint. [Version 1] doi: 10.64898/2026.03.28.714908 (PMC13060065; doi:10.64898/2026.03.28.714908)
Supplement: Supplementary file 1 [file NIHPP2026.03.28.714908v1-supplement-1.pdf]

bioRxiv preprint doi: https://doi.org/10.64898/2026.03.28.714908; this version posted March 30, 2026. The copyright holder for this preprint (which was not certified by peer review) is the author/funder, who has granted bioRxiv a license to display the preprint in perpetuity. It is made available under aCC-BY 4.0 International license.

Supplementary Figure 1. MCM8 expression analysis.

(A) Gel electrophoresis of *Mcm8* and *Actb* RT-PCR products from adult testes (primer set 1 in **Figure 2A**; quantified in **Figure 2C**). (**B**) Immunoblot of MCM8 and VINCULIN (loading control) in adult whole-testis extracts. Relative MCM8 signal intensities normalized to VINCULIN are noted.

Supplementary Figure 2. Analysis of juvenile meiosis.

(A) Representative images of juvenile (16 dpp) seminiferous tubules immunostained for SYCP3 and γH2AX. (**B**) Bar plots show distribution of seminiferous tubule types in individual mice of the indicated ages. Tubule types (diagrammed on the right) were categorized based on presence of leptotene or zygotene (L/Z) and pachytene (P) stages, as judged by SYCP3- and γH2AX-staining patterns.

Supplementary Figure 3. Altered recombination foci in *Mcm8* mutants.

(A) Chromosome spreads depicting time course of RAD51 staining during meiotic prophase. (**B**) Chromosome spreads depicting time course of RPA2 staining across meiotic prophase.

Supplementary Figure 4. γH2AX time course in *Spo11* mutants.

γH2AX intensity measurements per cell, normalized to the mean intensity of wild-type leptotene stage cells. Data from **Figure 6 (D)** was replotted with an expanded y-axis to better visualize differences between *Mcm8*$^{+/+}$ and *Mcm8*$^{m/m}$ mice.

Supplementary Figure 5. Analysis of resection in *Mcm8* mutants.

(**A**) Correlation between DMC1 SSDS read counts (reads per million; RPM) at B6 hotspots present in adult *Mcm8*$^{m/m}$ and wild type-littermates. (**B**) Metaplots of DMC1 and RPA2 SSDS averages around B6 wild-type hotspots. Reads originating only from the reverse (rev) strands

bioRxiv preprint doi: https://doi.org/10.64898/2026.03.28.714908; this version posted March 30, 2026. The copyright holder for this preprint (which was not certified by peer review) is the author/funder, who has granted bioRxiv a license to display the preprint in perpetuity. It is made available under aCC-BY 4.0 International license.

are shown for clarity. Red arrow in wild type points to RPA-SSDS reads that have the wrong polarity to be resection tracts. (**C**) Distribution of resection tract lengths relative to B6 wild-type hotspots. Fractions of total reads were calculated every 100 bp and plotted. Mean resection lengths are indicated. (**D**) Heatmaps of strand-specific S1-seq reads counts around B6 wild-type hotspots in adult mice. Each row is a single hotspot, ranked from the strongest at the top.

Supplementary Figure 6. MCM8 binding to oligonucleotide-based DNA structures.

(**A**) Recombinant MCM8-9 complex and MCM8 alone used for the biochemical experiments. The polyacrylamide gel was stained with Coomassie Brilliant Blue. (**B**) Representative gels for binding assays (quantified in **Figure 8**). Schematics of substrates used are shown on the top and red asterisks represent the positions of the radioactive labels.

bioRxiv preprint doi: https://doi.org/10.64898/2026.03.28.714908; this version posted March 30, 2026. The copyright holder for this preprint (which was not certified by peer review) is the author/funder, who has granted bioRxiv a license to display the preprint in perpetuity. It is made available under aCC-BY 4.0 International license.

Supplementary Figure 1

A

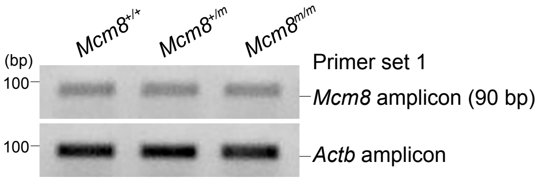

B

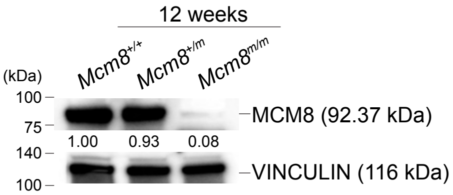

bioRxiv preprint doi: https://doi.org/10.64898/2026.03.28.714908; this version posted March 30, 2026. The copyright holder for this preprint (which was not certified by peer review) is the author/funder, who has granted bioRxiv a license to display the preprint in perpetuity. It is made available under aCC-BY 4.0 International license.

Supplementary Figure 2

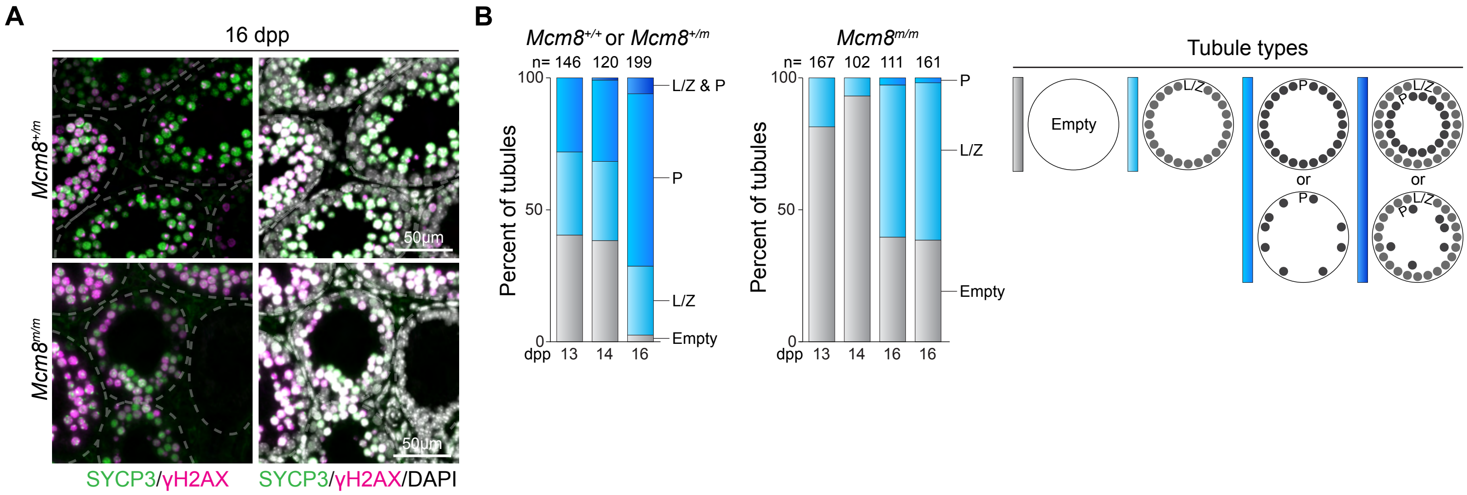

bioRxiv preprint doi: https://doi.org/10.64898/2026.03.28.714908; this version posted March 30, 2026. The copyright holder for this preprint (which was not certified by peer review) is the author/funder, who has granted bioRxiv a license to display the preprint in perpetuity. It is made available under aCC-BY 4.0 International license.

Supplementary Figure 3

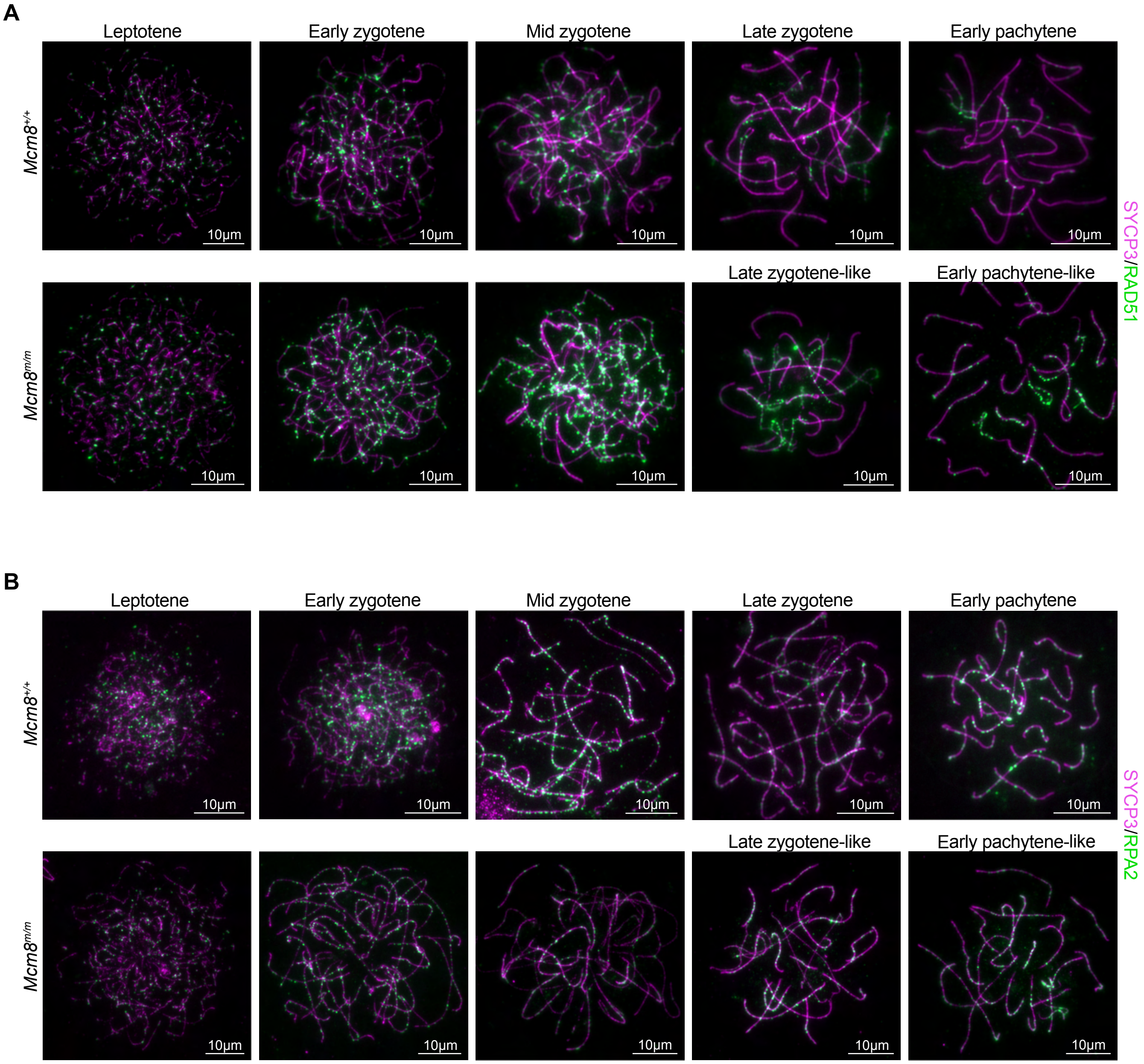

bioRxiv preprint doi: https://doi.org/10.64898/2026.03.28.714908; this version posted March 30, 2026. The copyright holder for this preprint (which was not certified by peer review) is the author/funder, who has granted bioRxiv a license to display the preprint in perpetuity. It is made available under aCC-BY 4.0 International license.

Supplementary Figure 4

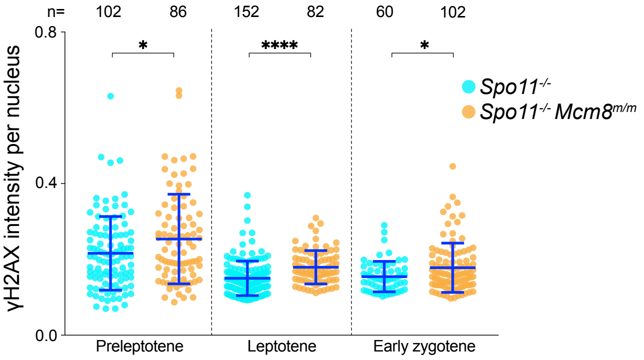

bioRxiv preprint doi: https://doi.org/10.64898/2026.03.28.714908; this version posted March 30, 2026. The copyright holder for this preprint (which was not certified by peer review) is the author/funder, who has granted bioRxiv a license to display the preprint in perpetuity. It is made available under aCC-BY 4.0 International license.

Supplementary Figure 5

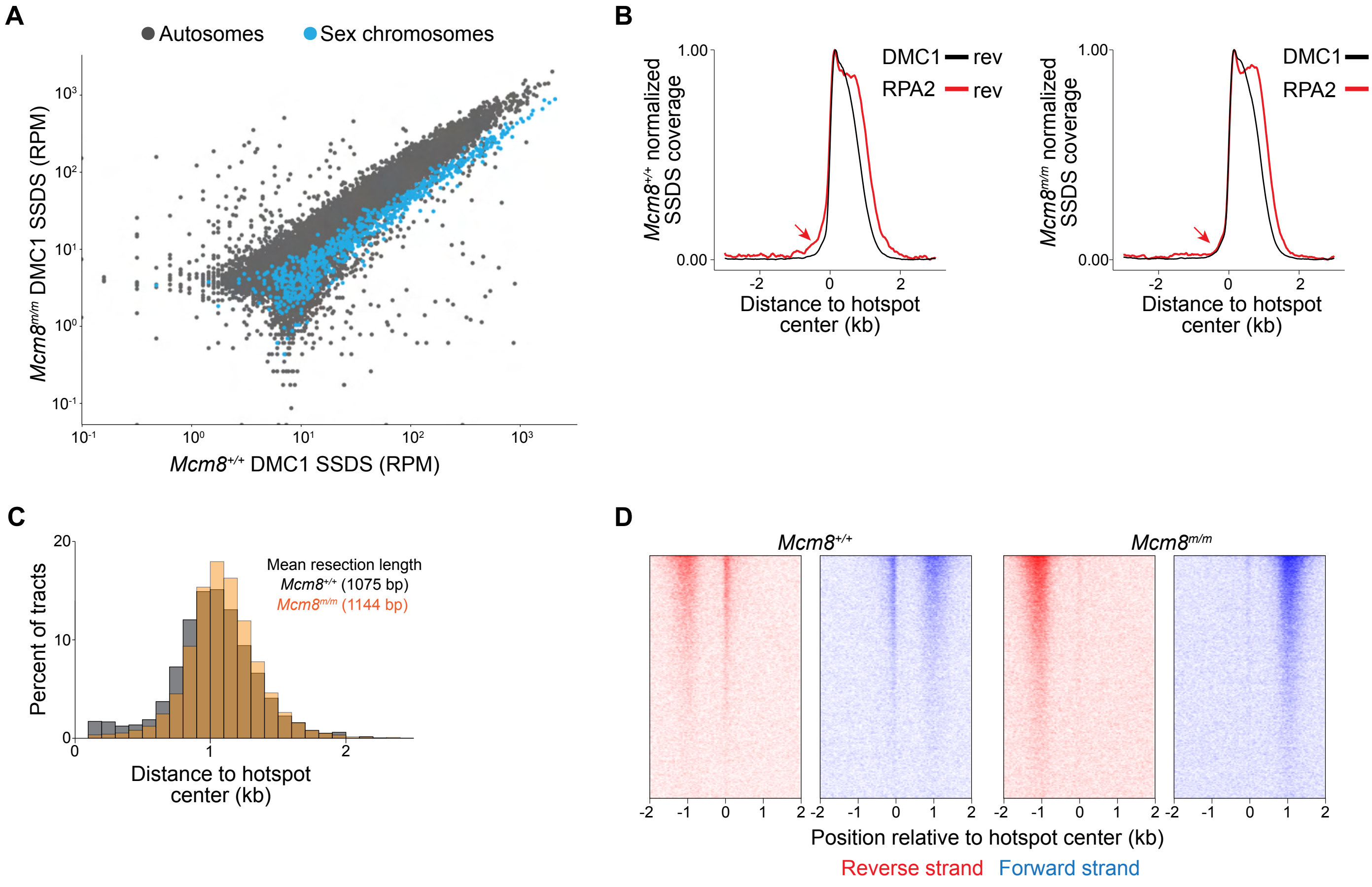

bioRxiv preprint doi: https://doi.org/10.64898/2026.03.28.714908; this version posted March 30, 2026. The copyright holder for this preprint (which was not certified by peer review) is the author/funder, who has granted bioRxiv a license to display the preprint in perpetuity. It is made available under aCC-BY 4.0 International license.

Supplementary Figure 6

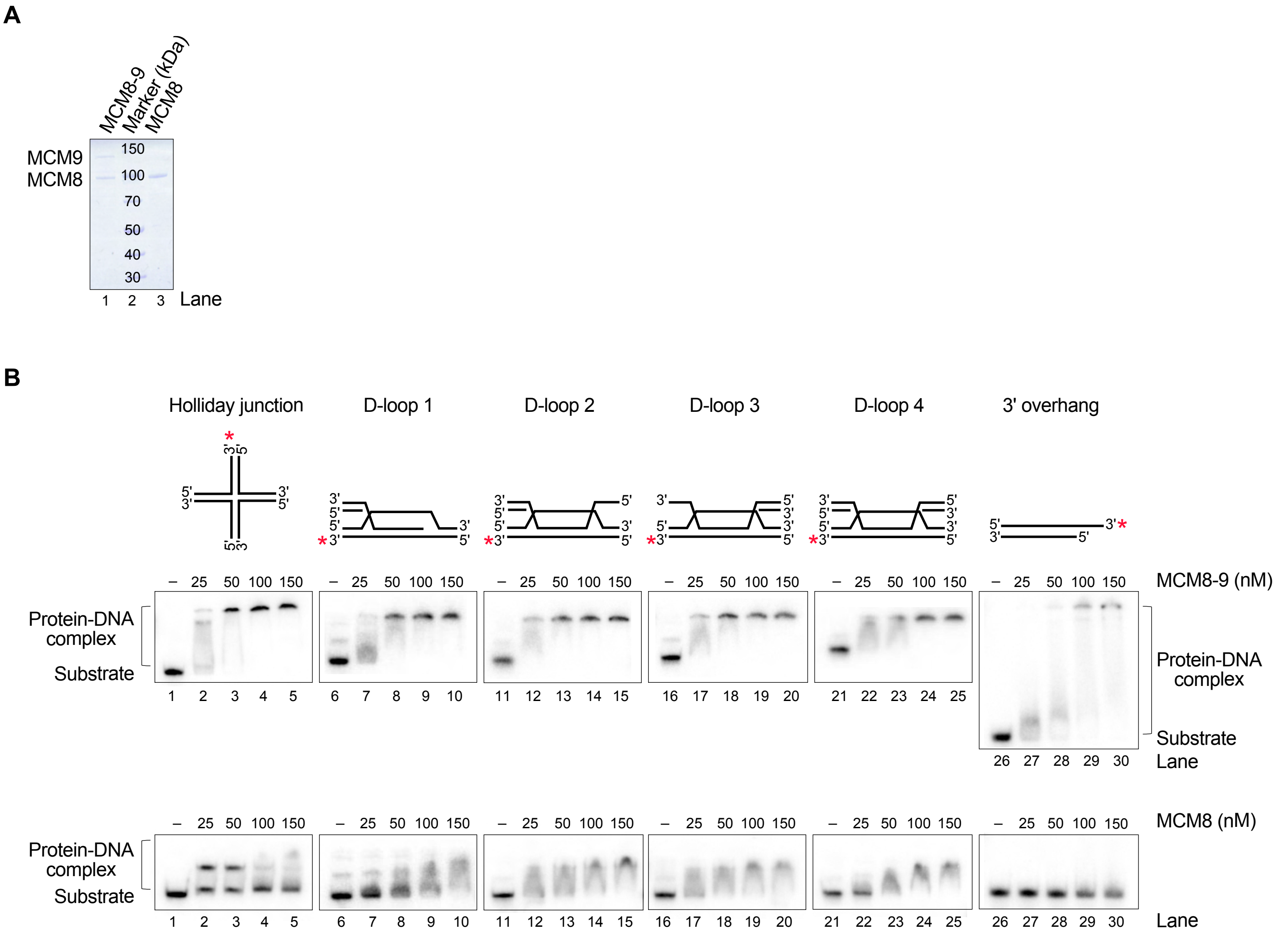

Supplementary Table 1. Antibodies.

Primary Antibody	Company	Reference	Host Species	Application	Concentration
DMC1	ProteinTech	13714-1-AP	Rabbit	Spread	1:100
DMC1	Gift from Florencia Pratto		Rabbit	Spread; SSDS	1:50; 2 µg
γH2AX	Novus Biological	NB100-384	Rabbit	Spread; Histology	1:1000; 1:250
MSH5	Gift from Florencia Pratto		Rabbit	Spread	1:50
MLH1	Cell Signaling	3515S	Mouse	Spread	1:25
MCM8	ProteinTech	16451-1-AP	Rabbit	Section; WB	1:100; 1:2500
RAD51	Millipore-Sigma	pc130	Rabbit	Spread	1:100
RPA2	Abcam	ab10359	Mouse	SSDS	2 µg
RPA2	Cell Signaling	2208S	Rat	Spread	1:75
STRA8	Abcam	ab49602	Rabbit	Histology	1:1000
SYCP1	Abcam	ab15090	Rabbit	Spread	1:100
SYCP3	Novus Biological	NB300-232	Rabbit	Spread	1:200
SYCP3	Abcam	ab15093	Rabbit	Spread	1:200
SYCP3	Santa Cruz	sc-74569	Mouse	Spread; Histology	1:200; 1:250
VINCULIN	Santa Cruz	sc-73614	Mouse	WB	1:2500
**Secondary Antibody**	**Company**	**Reference**	**Host Species**	**Application**	**Concentration**
Mouse 488	Invitrogen	A21202	Donkey	Spread; Histology	1:250; 1:500
Mouse 594	Invitrogen	A11032	Goat	Spread; Histology	1:250; 1:500
Mouse Digital	Kindle Biosciences	R1005		WB	1:2000
Rabbit 488	Invitrogen	A11034	Goat	Spread; Histology	1:250; 1:500
Rabbit 594	Invitrogen	A21207	Donkey	Spread; Histology	1:250; 1:500
Rabbit Digital	Kindle Biosciences	R1006		WB	1:1000
Rat 488	Invitrogen	A11006	Goat	Spread	1:250

Supplementary Table 2. Oligonucleotides used for preparation of DNA substrates.

Name	Sequence
PC1255	5'-gtcggatcctctagacagctccatgatcactggcactggtagaattcggc-3'
314	5'-catggagctgtctagaggatccgac-3'
PC1253	5'-tgggtcaacgtgggcaaagatgtcctagcaatgtaatcgtctatgacgtt-3'
PC1254	5'-tgccgaattctaccagtgccagtgatggacatctttgcccacgttgaccc-3'
PC1256	5'-caacgtcacagacgattacattgctacatggagctgtctagaggatccga-3'
BB	5'-tcaagctcggtctgcagtcaggatgattgtgagcgttaaccctaaccctaaccctaaccctaatctgcactcgagactcacgtcctggtcacg-3'
BT	5'-cgtgaccaggacgtgagtctcgagtgcagaccttttttttttttttttttttttttttttttttacaatcatcctgactgcagaccgagcttga-3'
InvA	5'-caccatccagttctcttcgcggc-3'
InvB	5'-gccgcgaagagaactggatggtg ttagggttagggttagggttagggttaacgctc-3'
InvD	5'-gccgcgaagagaactggatggtgttagggttagggttagggttagggttaacgctcct cacttccaccgcagaaggtgg-3'
InvE	5'-ccaccttctgcggtggaagtgag-3'
